# Alterations of endocannabinoids in cerebrospinal fluid of dogs with epileptic seizure disorder

**DOI:** 10.1186/1746-6148-9-262

**Published:** 2013-12-26

**Authors:** Felix K Gesell, Alexander A Zoerner, Christina Brauer, Stefan Engeli, Dimitros Tsikas, Andrea Tipold

**Affiliations:** 1 Department of Small Animal Medicine and Surgery, University of Veterinary Medicine, Hannover, Germany; 2 Institute for Clinical Pharmacology, Hannover Medicine School, Hannover, Germany

**Keywords:** Endocannabinoids, Anandamide, 2-arachidonyl glycerol, Epilepsy, Cerebrospinal fluid, Canine

## Abstract

**Background:**

Epilepsy is one of the most common chronic neurological disorders in dogs characterized by recurrent seizures. The endocannabinoid (EC) system plays a central role in suppressing pathologic neuronal excitability and in controlling the spread of activity in an epileptic network. Endocannabinoids are released on demand and their dysregulation has been described in several pathological conditions. Recurrent seizures may lead to an adverse reorganization of the EC system and impairment of its protective effect. In the current study, we tested the hypothesis that cerebrospinal fluid (CSF) concentrations of the endocannabinoids anandamide (AEA) and 2-arachidonoyl glycerol (2AG) are altered in epileptic dogs. Concentrations of AEA and total AG (sum of 2AG and 1AG) were measured in 40 dogs with idiopathic epilepsy and in 16 unaffected, healthy control dogs using liquid chromatography combined with tandem mass spectrometry.

**Results:**

AEA and total AG were measured at 4.94 (3.18 – 9.17) pM and 1.43 (0.90 – 1.92) nM in epileptic dogs and at 3.19 (2.04 – 4.28) pM and 1.76 (1.08 – 2.69) nM in the control group, respectively (median, 25 – 75% percentiles in brackets). The AEA difference between epileptic and healthy dogs was statistically significant (p < 0.05). Values correlated with seizure severity and duration of seizure activity. Dogs with cluster seizures and/or status epilepticus and with seizure activity for more than six months displayed the highest EC concentrations.

**Conclusion:**

In conclusion, we present the first endocannabinoid measurements in canine CSF and confirm the hypothesis that the EC system is altered in canine idiopathic epilepsy.

## Background

Epilepsy is one of the most common neurological disorders in dogs, characterized by recurrent seizures [1,2]. Based on underlying etiology epilepsy in dogs can be diagnosed as idiopathic or symptomatic [2,3]. In idiopathic epilepsy, hereditary factors are responsible for recurrent seizures [4]. Seizures reflect an abnormal hypersynchronous electrical activity of neurons, caused by an imbalance between excitation and inhibition in the brain [1]. Most dogs with idiopathic epilepsy suffer their first seizure between one and five years of age and although any breed - including mix-breeds - can be affected, a genetic basis for idiopathic epilepsy is suggested for a number of breeds [5]. The prevalence of epilepsy in dogs has been estimated in different studies to vary from 0.5 to 5% [1].

Using current treatment protocols a significant part of epileptic dogs may still continue to suffer from seizures [4-7]. Better understanding of the molecular pathogenesis of seizure development would allow introducing new treatment modalities.

The endocannabinoid (EC) system displays numerous physiological functions [8]. Anandamide (AEA) and 2-arachidonylglycerol (2AG), the two most studied endocannabinoids, are endogenous lipid mediators that bind to the G protein coupled cannabinoid receptors type 1 and type 2 (CB1 and CB2). CB1 is one of the most abundant receptors in the mammalian brain and also present in peripheral tissues [9]. Similary, CB2 is expressed in various tissues, especially on cells of the immune system [10]. Endocannabinoids are involved in food intake, pain sensation and memory formation, amongst others [8,11]. EC system dysregulation is connected to several pathological conditions [10], such as Alzheimer’s, Parkinson’s, and Huntington’s disease, obesity, ischemic brain damage and epileptic seizures [12-15]. Endocannabinoids are released from the postsynaptic neurons and serve as retrograde signaling molecules [16]. Generally, retrograde endocannabinoid signaling leads to decreased synaptic transmission and might therefore be involved in maintaining the seizure threshold. Consequently, endocannabinoid signaling at glutamatergic synapses could have a beneficial effect in epilepsy treatment [14]. The exact role of endocannabinoids in epilepsy pathophysiology is still unclear. A recent study found significant decreased AEA concentrations in cerebrospinal fluid (CSF) of human epileptic patients with newly diagnosed seizures as compared to healthy controls. No differences in 2AG concentrations were observed [17]. In another study, 2AG hippocampal tissue concentrations were increased in a rat model of pilocarpine-induced status epilepticus compared to control animals. The authors concluded that seizure activity in otherwise healthy rats increases endocannabinoid synthesis, possibly as a protective mechanism [18].

Although dogs appear to be an ideal model for the study of human epileptic seizures [19], endocannabinoids have never been measured in canine CSF. We therefore hypothesized that altered CSF concentrations of AEA and 2AG are involved in the process of canine epilepsy. In order to prove it, AEA and total arachidonyl glycerol (total AG, i.e. the sum of 2AG and 1AG) in CSF of 40 dogs with idiopathic epilepsy and in a control group of 16 unaffected healthy dogs were measured. Correlations between AEA and seizure severity and disease duration were calculated.

## Results

**Figure 1:**
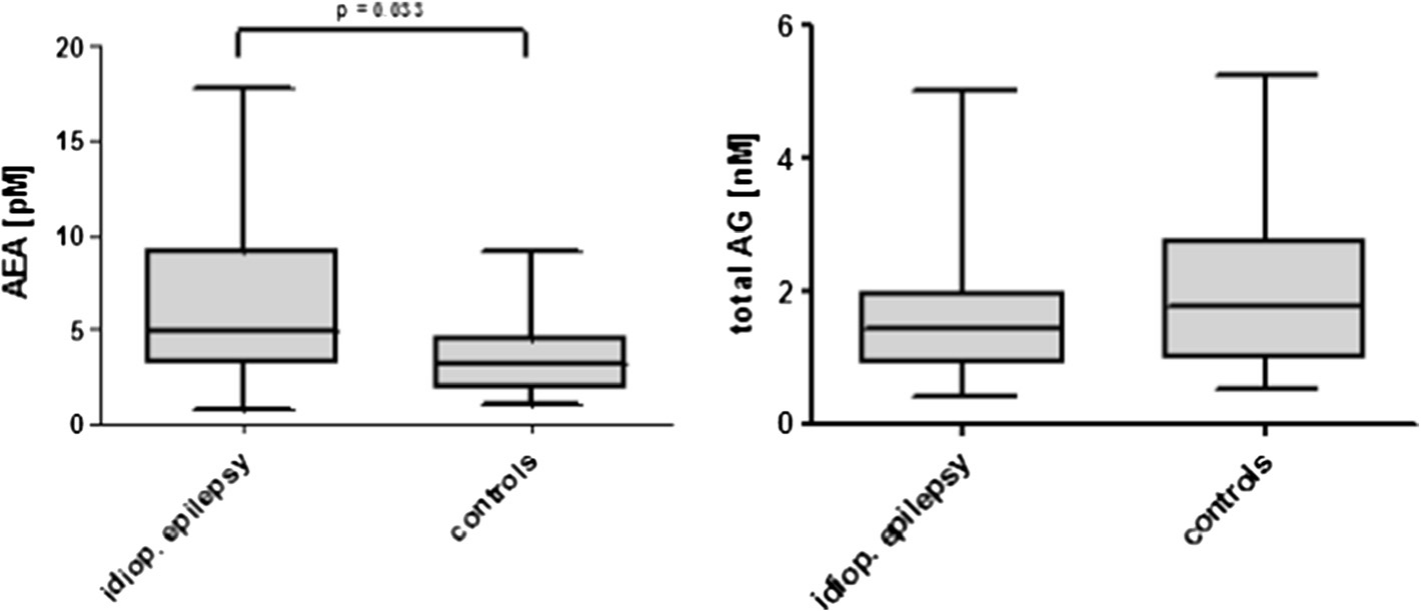
AEA and total AG concentrations of 40 dogs with idiopathic epilepsy and 16 control dogs, statistic was calculated using the Wilcoxon-Test, central lines of the box represent the median, upper and lower limits of the box represent the 75 th and 25 th percentiles.

AEA and total AG were detected and quantified in nearly all of the CSF samples. Well in line with previous endocannabinoid measurements in CSF and brain tissue [17,20,21], concentrations were in the picomolar range for AEA and in the nanomolar range for total AG. Animals suffering from idiopathic epilepsy had higher AEA concentrations than control animals (median, 25 – 75% percentiles: 4.94, 3.18 – 9.17 pM vs. 3.19, 2.04 – 4.28 pM, p = 0.033) (Table 1). No statistically significant difference was observed for total AG concentration (Figure 1).

**Table 1:** AEA levels and comparison of different subgroups

**Groups**	**Number of dogs**	**AEA levels (pM)**	**p -values**
Dogs with idiopathic epilepsy (IE)	40	4.94 (3.18– 9.17)	p = 0.033
Control group	16	3.19 (2.04 – 4.28)	
Dogs with history of cluster seizures and/or status epilepticus	23	8.05 (4.18 – 12.80)	p = 0.029
Dogs with single generalized seizures	16	3.81 (1.77 – 6.41)	
Dogs with IE for > 6 months	16	8.30 (4.60 – 15.00)	p = 0.036
Dogs with IE for < 6 months	20	4.10 (2.99 – 7.85)	

IE, Idiopathic epilepsy.

Group differences of endocannabinoid concentrations were tested using the Wilcoxon-Test.

Differences were considered statistically significant when the p value was < 0.05.

AEA levels are presented as median and 25 – 75% percentiles.

**Figure 2:**
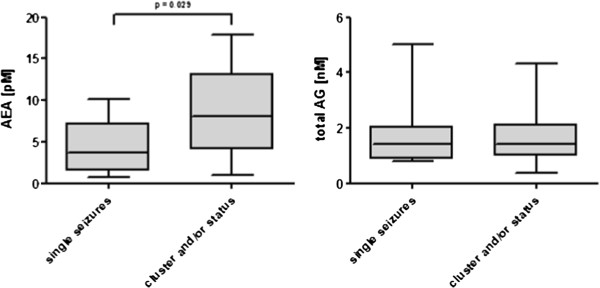
AEA and total AG concentrations of 16 dogs with single seizures and 23 dogs with cluster seizures and/or status epilepticus, statistic was calculated using the Wilcoxon-Test, central lines of the box represent the median, upper and lower limits of the box represent the 75 th and 25 th percentiles.

In a subgroup of dogs with severe seizures (n = 23, history of cluster seizures and/or status epilepticus), significantly higher AEA concentration in CSF were measured (median, 25 – 75% percentiles: 8.05, 4.18 – 12.80 pM) than in dogs with single seizure episodes (n = 16, no cluster seizures and no status epilepticus, median, 25 – 75% percentiles: 3.81, 1.77 – 6.41 pM, p = 0.029). No significant difference in total AG was observed (Figure 2).

**Figure 3:**
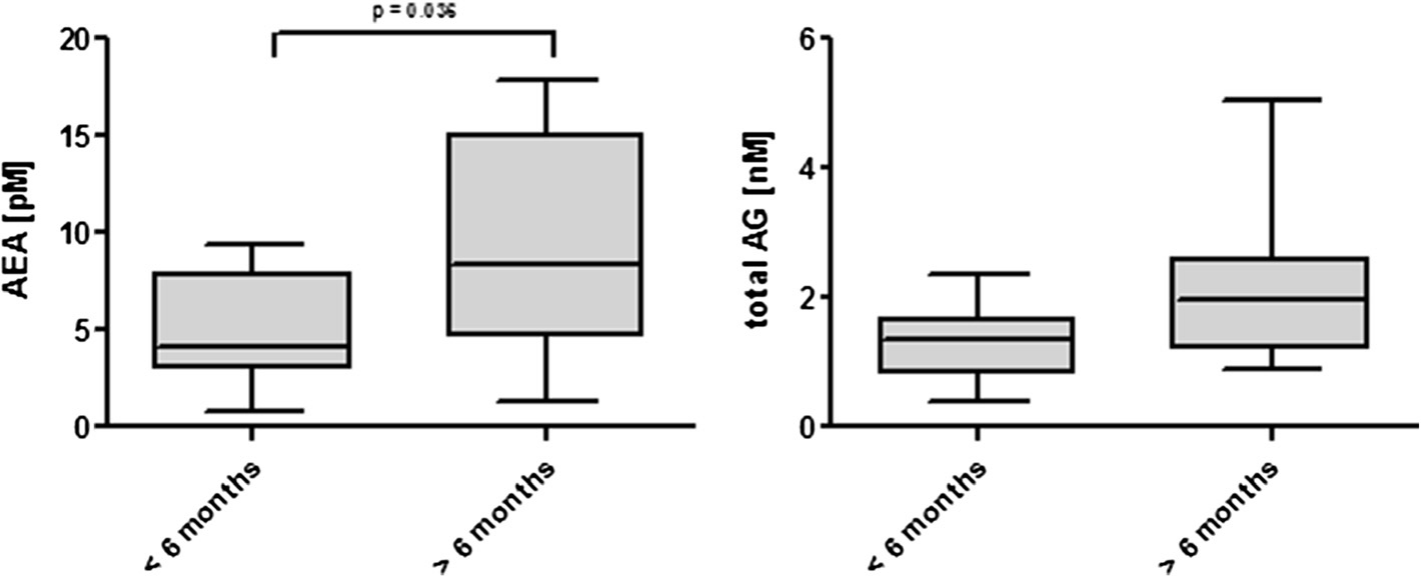
AEA and total AG concentrations of 20 dogs with idiopathic epilepsy for less than 6 months and 16 dogs with idiopathic epilepsy for more than 6 months, statistic was calculated using the Wilcoxon-Test, central lines of the box represent the median, upper and lower limits of the box represent the 75 th and 25 th percentiles.

Besides seizure severeness, disease duration was also associated with higher AEA concentrations in CSF of dogs that suffered from idiopathic epilepsy for longer than six months (n = 16) presented with 8.30, 4.60 – 15.00 pM, whereas dogs with a history of idiopathic epilepsy for shorter than six months (n = 20) presented with 4.10, 2.99 – 7.85 pM (p = 0.036). Total AG concentrations in these subgroups were again not significantly different (Figure 3). Other calculated correlations included age of the dogs, pretreated and untreated patients and timepoint of CSF collection and did not reveal statistical significant findings. However, the number of patients in these subgroups was limited.

## Discussion

Although epilepsy is one of the most common neurological diseases in dogs, a seizure free life cannot be achieved in many patients by current treatment options. Using well known antiepileptic drugs, approximately 25% of dogs cannot be well controlled and continue to have seizures [5,7]. Thus, in the authors opinion new treatment options are needed and the controlled therapeutic modulation of the EC system could represent one possibility in order to treat those refractory epileptic patients. Therefore, pathophysiological mechanisms of the EC system have to be better understood.

EC system dysregulation appears to play a role in the process of epilepsy [13]. In the central nervous system (CNS), endocannabinoids are released on demand from membrane phospholipid precursors at postsynaptic neurons and bind to G-protein coupled cannabinoid receptors at the presynaptic neuron after retrograde diffusion [10]. The following signal cascade involves inhibition of cyclase activity or different types of calcium channels and activation of certain potassium channels [9]. Overall, endocannabinoid release leads to decreased synaptic transmission and might therefore be involved in maintaining the seizure threshold [14].

In a human study examining newly-diagnosed epileptic patients, CSF concentrations of AEA were reduced significantly in affected patients compared to healthy controls [17]. In contrast, AEA concentrations in affected dogs were increased compared to the control group. We not only studied newly-diagnosed epileptic dogs but also dogs with a longer history of epilepsy. AEA concentrations in dogs with a seizure-onset more than six months prior to CSF-sampling were increased compared to those dogs with a seizure-onset within the last six months. We therefore suggest that EC system changes occur slowly and represent a counterregulatory mechanism to the pathological changes associated with epilepsy. The fact that only newly-diagnosed patients were included in the human study [17] may explain the contradictory findings. A limitation of the current study is that the number of patients was limited in the different subgroups.

In an *in vivo* study of neonatal rats with widespread neurodegeneration [20], the authors concluded that pathological events provide a stimulus for anandamide formation, finally leading to increased AEA concentrations. This interpretation supports our findings of increased AEA concentrations in the CSF of epileptic dogs.

Not only the concentration of endocannabinoids but also the density of the endocannabinoid receptors seems to be altered in the CNS of epileptic patients. CB1 receptor mRNA expression was down regulated in the human hippocampus of epileptic patients compared to healthy controls [22]. This down regulation confirms the hypothesis that protective endocannabinoid signalling is diminished in epileptic patients and may result in the incapability of increased AEA concentrations to inhibit pathological hyperexcitability.

In another study with pilocarpine-induced epilepsy in mice, CB1-receptor down regulation in the hippocampus was only found in the acute phase, whereas in the chronic phase, an upregulation was observed [8].

Dogs with severe seizures including cluster seizures and/or status epilepticus, had significant higher AEA CSF concentrations compared to dogs with single seizure events. We suggest that severe seizures increase cellular AEA release in order to control these seizures. If elevated AEA concentrations and an activation of the EC system were partially responsible for the disease, elevated AEA concentrations would have been expected to occur in all epileptic dogs including dogs with single seizure events.

Disease duration also influenced CSF endocannabinoid concentrations. A higher concentration of AEA was measured in the CSF of dogs that suffered from idiopathic epilepsy for longer than six months compared to the dogs with idiopathic epilepsy for less than six months. We suggest that more AEA is released over time in order to control the seizures and therefore dogs with longer disease duration show higher CSF endocannabinoid concentrations. We would expect elevated AEA concentrations to occur in all epileptic dogs including newly diagnosed dogs, if elevated AEA concentrations and an activation of the EC system contributed to the development of the disease. Despite the observed elevated AEA concentration in animals suffering for less than six months from epilepsy compared to the control animals, the further increase of AEA over time suggests a counterregulation of the EC system or seizure activity itself is leading to AEA increase.

Another interesting aspect would be the exact time elapsing between the last seizure and CSF sampling. Because of the retrospective character of our study it was not possible to investigate this aspect. We would hypothesize that immediately after the seizure event the endocannabinoid concentration is at its highest point. Such a suggestion would support our results and our hypothesis that ECs are released as a counterregulatory mechanism in order to control prolonged seizure events.

## Conclusion

In conclusion, we demonstrated an elevation of CSF AEA concentrations in dogs with idiopathic epilepsy. The highest AEA concentrations were found in dogs with severe seizures and a long disease history. Possibly, the activation of the EC system serves as a counter-mechanism in order to regulate the seizure-threshold in epilepsy. However, the EC system can either alter or be altered by seizure activity, so that further, prospective studies are warranted to investigate pathological mechanisms. Despite endocannabinoids can be synthesized “on demand”, the EC system should be considered for development of new treatment strategies against epilepsy.

## Methods

This retrospective analysis included data and CSF samples of patients of the Department of Small Animal Medicine and Surgery of the University of Veterinary Medicine Hannover collected between January 2010 and February 2012. The study was conducted in accordance with the ethical rules of the university and approved by the national authority (number 33.9-42502-05-12A214). Fourty dogs with idiopathic epilepsy and 16 healthy dogs were included in this study. The group of dogs with idiopathic epilepsy included 19 females (13 were neutered) and 21 males (7 were sterilized), ranged between 10 months and 11 years of age (median of 3 years). The group included 26 different breeds dominating mix breed (6/40) and Labrador retriever (4/40). The healthy group included 4 females (1 was neutered) and 12 males (1 was sterilized), ranged between 7 months and 8 years of age (median of 1 ½ years). The group included 7 different breeds dominating the Beagles (9/16).

CSF samples without blood contamination had been collected from all patients, frozen and stored at −20°C until analysis. Patient history was analyzed from clinical records and included age at epilepsy onset, seizure frequency, previous therapy and information about occurrence of status epilepticus or cluster seizures. Idiopathic epilepsy had been diagnosed on the basis of routine clinical and neurological examination. No abnormalities on hemogram, chemistry profile and magnetic resonance imaging (MRI) were detected. All dogs with idiopathic epilepsy had routine CSF parameters in physiological ranges relating to the cell count (0 – 3 cells/μl), glucose (60 – 80% of the blood glucose concentration) and protein (less than 25 mg/dl) after suboccipital puncture [23]. Clinical and neurological examinations in all 16 healthy control dogs revealed no pathological findings and no history of seizures in the past.

AEA and total AG were measured by stable-isotope dilution liquid chromatography combined with tandem mass spectrometry (LC-MS/MS) as previously described [24]. To improve extraction efficacy, hydroxypropyl-β-cyclodextrine (10% w/v) was added after thawing the CSF samples on ice prior to liquid-liquid extraction. To 500 μl of each sample the internal standards d_4_-AEA and d_5_-2AG were added to a final concentration of 1.0 nM and 0.9 nM, respectively. Liquid-liquid extraction was performed by adding 500 μl toluene and shaking for 2 × 20 s at 5000 rpm in a PreCellyshomogenisator. After centrifugation (5 min, 4500 × g, 4°C) the organic phase was separated and evaporated to dryness by a gentle stream of nitrogen. The residues were reconstituted in 50 μl eluent for LC analysis (25% water, 75% methanol). 25 μl of the solution was injected into the Waters ACQUITY/XEVO TQ-MS LC-MS/MS system. Chromatographic separation took place on a Waters ACQUITY BEH C18 reversed phase column (100 mm × 2.1 mm ID, 1.7 μM particle size). The following transitions were monitored: *m/z*348 → *m/z *62 (AEA), *m/z*352 → *m/z*66 (d_4_-AEA), *m/z* 379 → *m/z* 287 (2AG), and *m/z* 384 → *m/z*287 (d_5_-2AG).

Because of the fast isomerization of 2AG to the biological inactive 1AG, quantification of 2AG is difficult to accomplish [21]. The available CSF samples in this study already contained a large portion of 1AG as compared to 2AG. Therefore, 2AG was calculated and referred to as the total AG concentration using the sum of the 2AG and 1AG peak areas in the acquired chromatograms.

Statistical analysis of data was performed by SAS 9.2. Due to non-gaussian data distribution as determined by the Shapiro-Wilk’s W test, group differences of endocannabinoid concentrations were tested using the Wilcoxon-Test. Differences were considered statistically significant when the p value was < 0.05. All data are presented as median and 25 – 75% percentiles.

## Competing interests

The authors declare that they have no competing interest.

## Authors’ contributions

All authors helped to draft the manuscript. FKG, AT and AAZ conceived of the study, and participated in its design and coordination. All authors read and approved the final manuscript.
